# Supplementary material for: Biophysical Characterization of Membrane Phase Transition Profiles for the Discrimination of Outer Membrane Vesicles (OMVs) From Escherichia coli Grown at Different Temperatures
Source: Front Microbiol. 2020 Feb 27;11:290. doi: 10.3389/fmicb.2020.00290 (PMC7056839; doi:10.3389/fmicb.2020.00290)

**Supplementary Materials**

**Biophysical characterization of membrane phase transition profiles for the discrimination of Outer Membrane Vesicles (OMVs) from Escherichia coli grown at different temperatures**

*Angelo Sarra, Antonella Celluzzi, Stefania Paola Bruno, Caterina Ricci, Simona Sennato, Maria Grazia Ortore, Stefano Casciardi, Federica Del Chierico, Paolo Postorino, Federico Bordi and Andrea Masotti*

**Figure S1.** Workflow of the procedures followed to isolate and purify bacterial vesicles for downstream measurements.

**Figure S2. (a), (c)** Dynamic Light Scattering intensity-weighed distribution of OMVs by *E. coli* grown at 27°C (a) and 20°C (c). **(b), (d)** Transmission Electron Microscopy image of vesicles by *E. coli* grown at 27°C (b) and 20°C (d) obtained without negative staining. In the insets are shown details of TEM images obtained with negative staining.


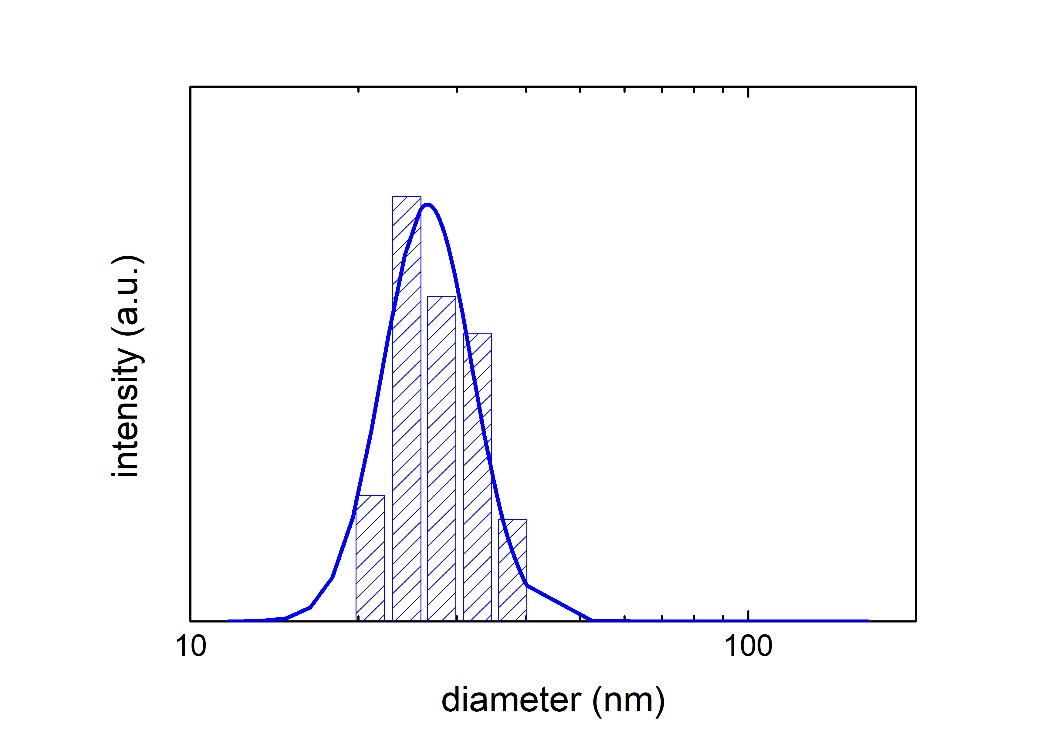

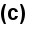

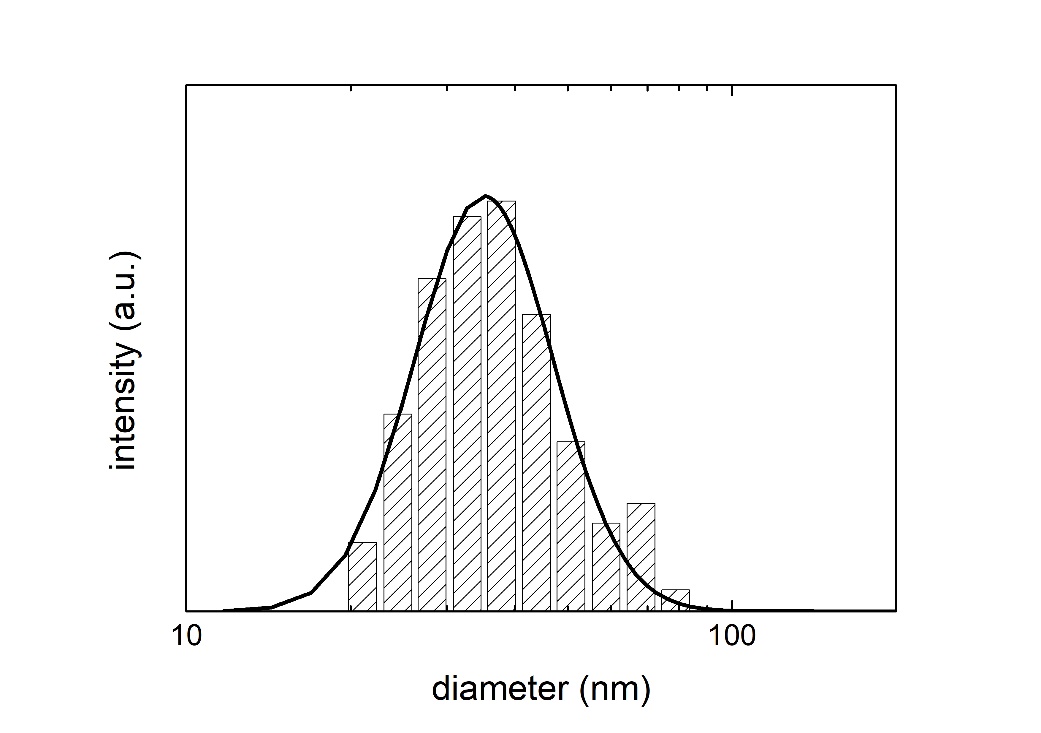

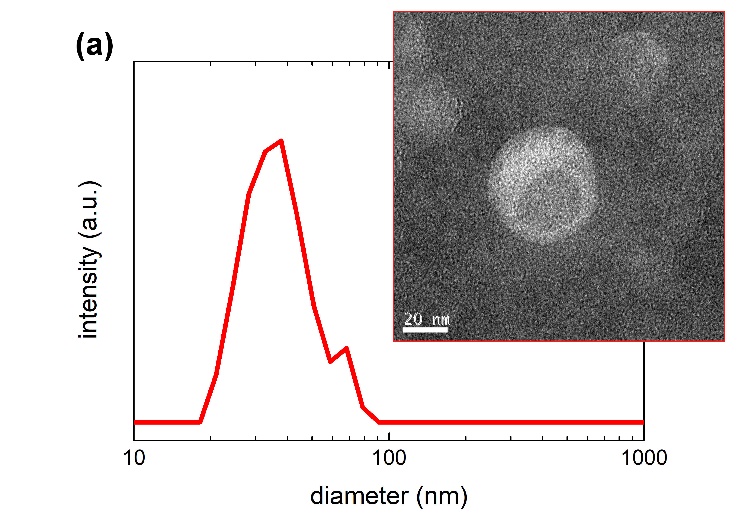

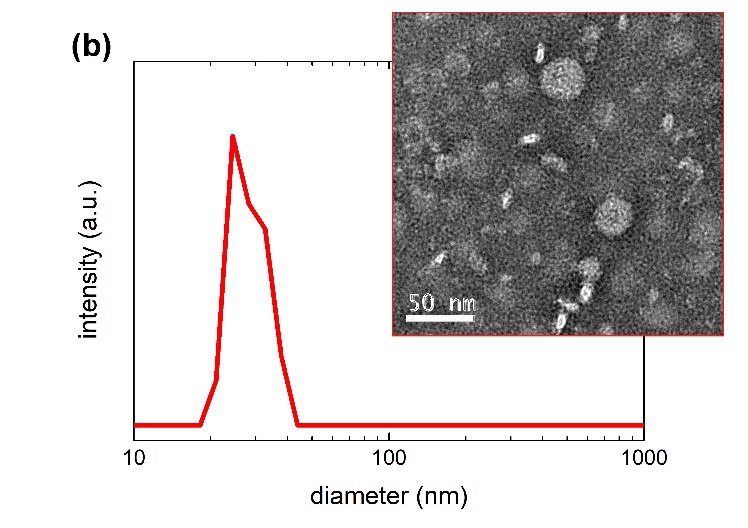

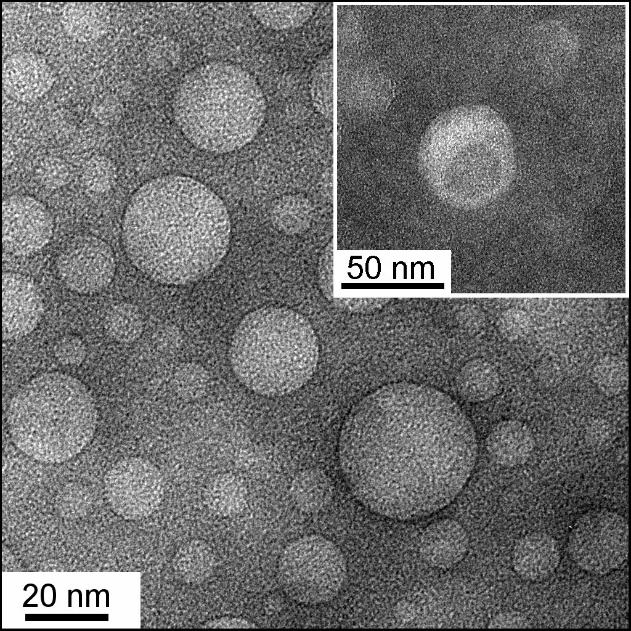

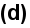

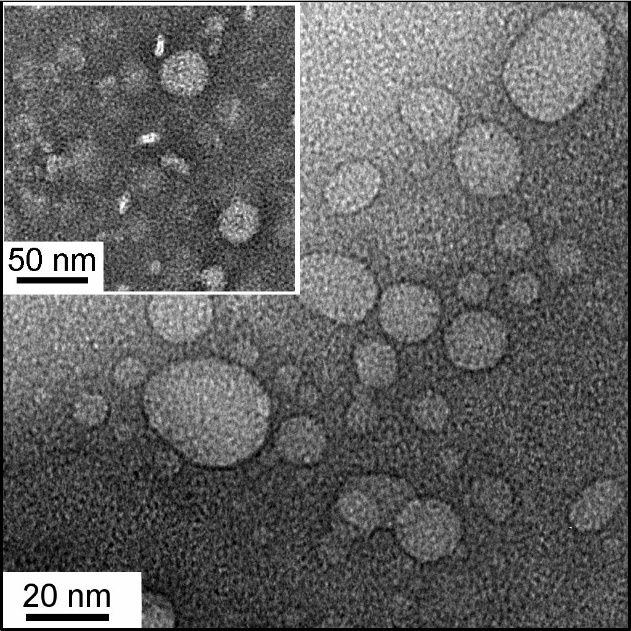


**Figure S3. (a)** Vesicles radius as resulting from SAXS data fitting procedure, as a function of temperature. **(b)** Vesicles core (black square) and shell (red circle) electron densities as resulting from SAXS data fitting procedure, as a function of temperature.


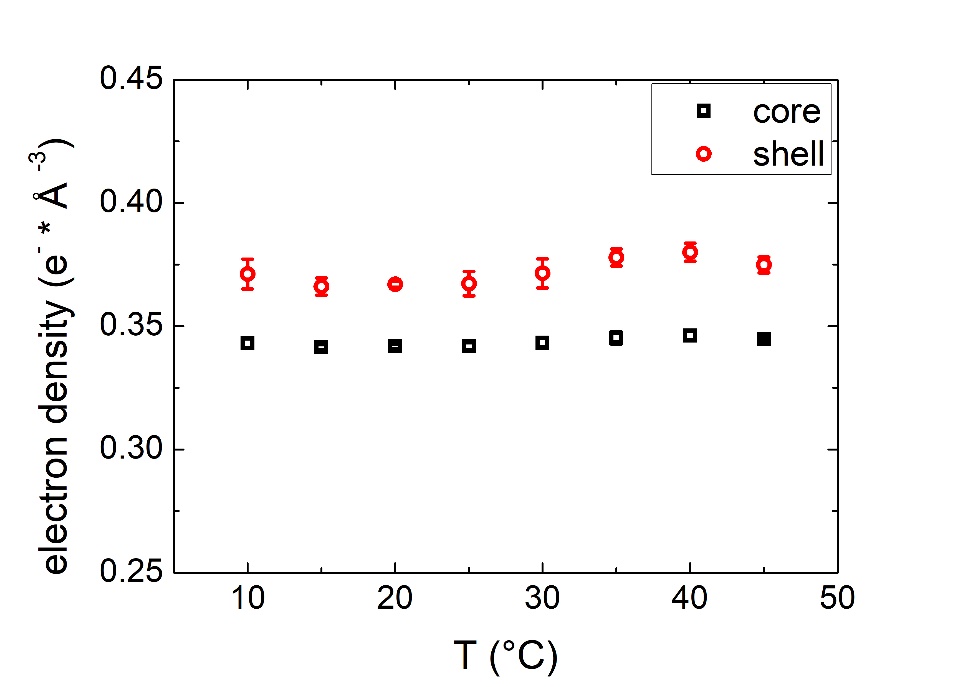

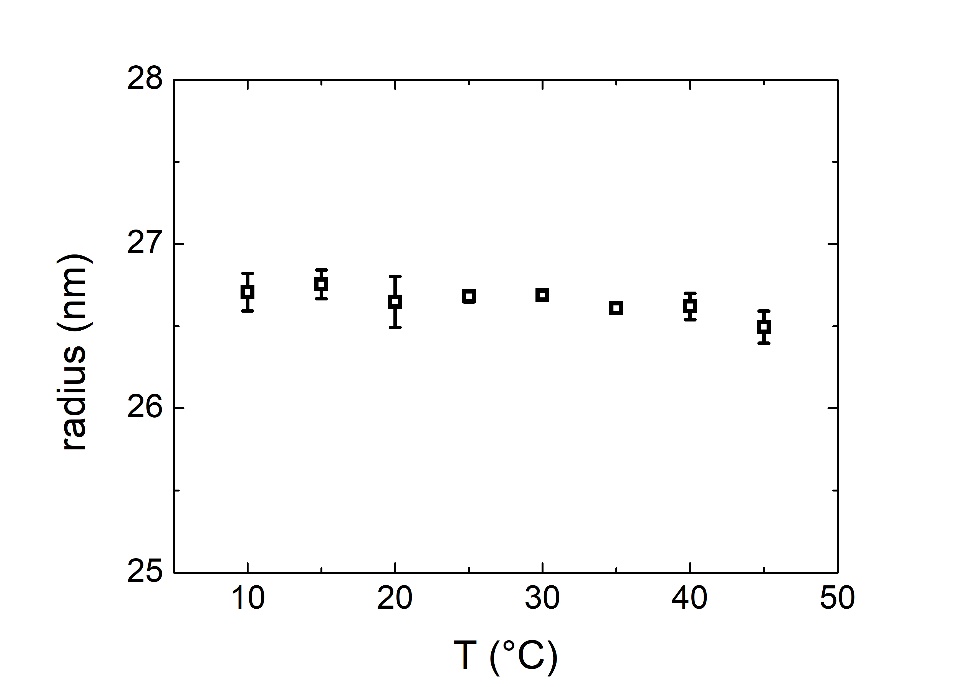

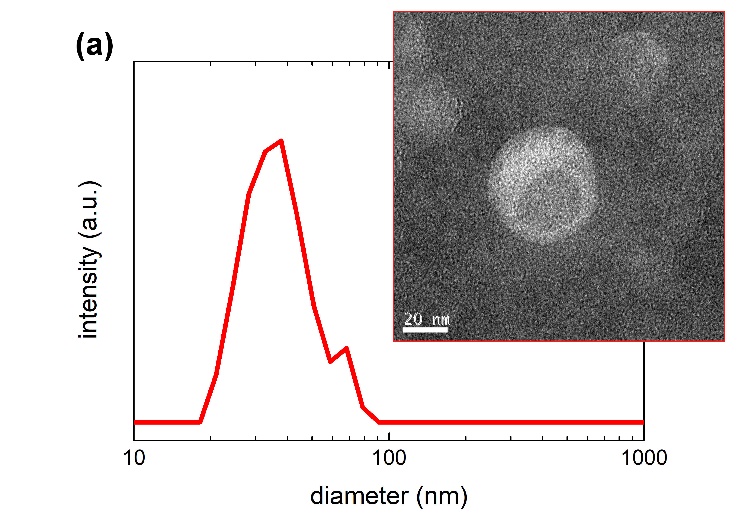

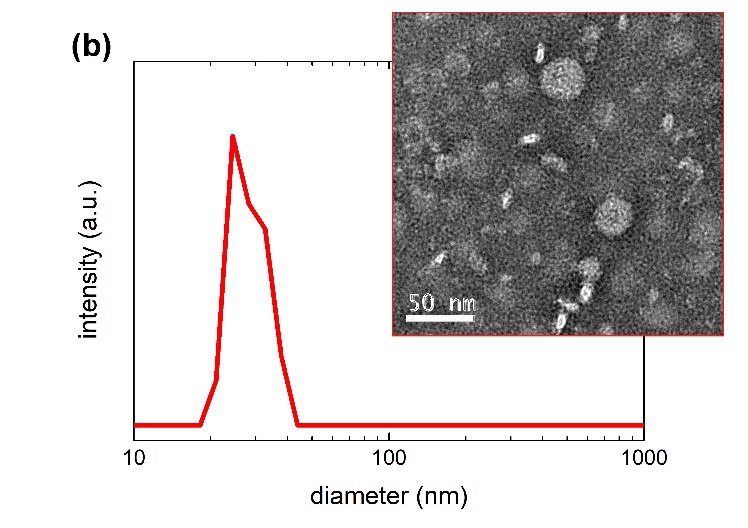


**Figure S4.** Hydrodynamic diameter of a DLS measurement at increasing temperatures between 10°C and 45°C of vesicles by *Lactobacillus rhamnosus* grown at 37°C.; red line result from linear fit.


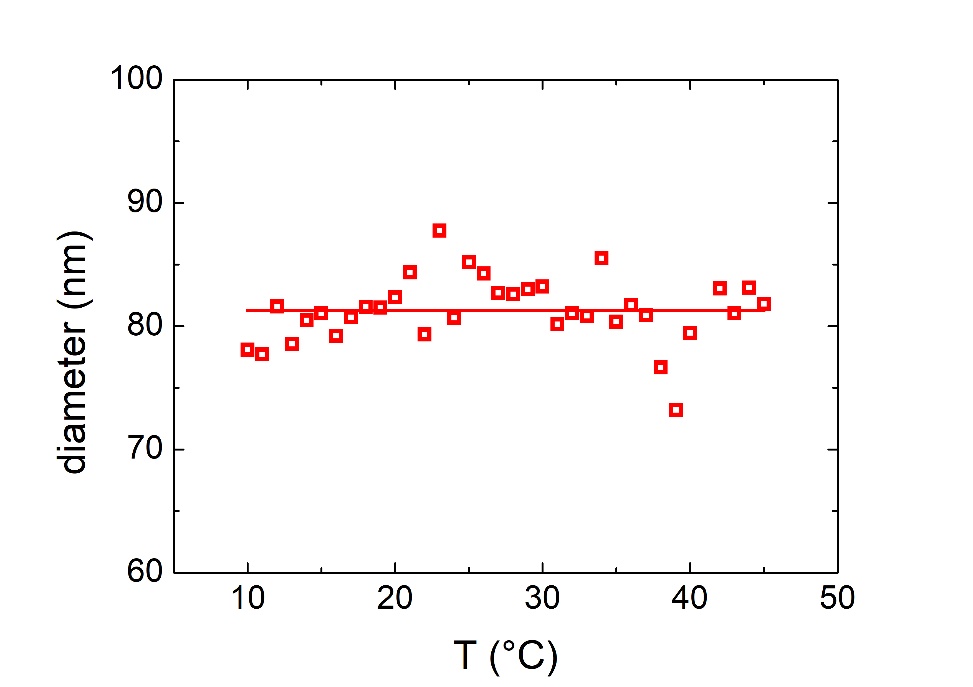

Supplement: Supplementary file 1 [file Data_Sheet_1.docx]
